# Iatrogenic Hypothalamus Injury After Resection of Subependymoma Within the Left Brain Ventricle

**DOI:** 10.7759/cureus.13044

**Published:** 2021-01-31

**Authors:** Jing Bao, Zhenjiang Pan, Shepeng Wei

**Affiliations:** 1 Neurosurgery, Shidong Hospital of Yangpu District in Shanghai, Shanghai, CHN

**Keywords:** iatrogenic hypothalamus injury, damage, diabetes insipidus, hyperthermia, acth deficiency

## Abstract

Damage to the hypothalamus may result from direct surgical injury or from hemorrhage and ischemia caused by the procedure. Patients with hypothalamus damage can be comatose and exhibit hyperthermia. Here, we present a patient whose hypothalamus was directly damaged by a drainage catheter. His clinical manifestations included diabetes insipidus, hyperthermia and adrenocorticotropic hormone (ACTH) deficiency. The patient was a 48-year-old male and had a body weight of 95 kg. He was admitted to the hospital on August 31, 2019 for memory impairment and nonspecific dizziness that persisted for four months. A magnetic resonance image of the head showed an intraventricular mass attached to the anterior third of the septum pellucidum and Monro's foramen and enlargement of the left lateral ventricle. This intraventricular cystic tumor was 1.9 cm in diameter, without gadolinium enhancement. The tumor resection was performed without complications and with less bleeding than expected. The patient developed central diabetes insipidus within just two hours after the operation and presented with hyperthermia within six hours after the operation. ACTH deficiency was evident on day 1 after surgery. After we removed the catheter 19 hours after the operation, the patient never developed polyuria or high fever again. Two months later, his ACTH level was normal and never needed to take prednisone again. This unusual complication should be taken into account in patients who need external ventricular drains. Much attention should be paid to ensure that the length of the drainage catheter beneath the brain surface does not exceed 5 cm.

## Introduction

The hypothalamus is an extremely important region of the central nervous system that is involved in temperature regulation, thirst and water balance, parturition and control of lactation, etc. [1].

Damage to the hypothalamus may result from direct surgical injury or from hemorrhage and ischemia caused by the procedure [2]. Injuries to the hypothalamus can likely result in death. Patients with these injuries can be comatose and exhibit hyperthermia [3].

Here, we present a patient whose hypothalamus was directly damaged by a drainage catheter. His clinical manifestations included diabetes insipidus, hyperthermia and adrenocorticotropic hormone (ACTH) deficiency. To our knowledge, this complication in patients who have undergone intraventricular tumor resection has not been previously reported in the literature.

## Case presentation

The patient was a 48-year-old male and had a body weight of 95 kg. He was admitted to the hospital on August 31, 2019 for memory impairment and nonspecific dizziness that persisted for 4 months.

**Figure 1:**
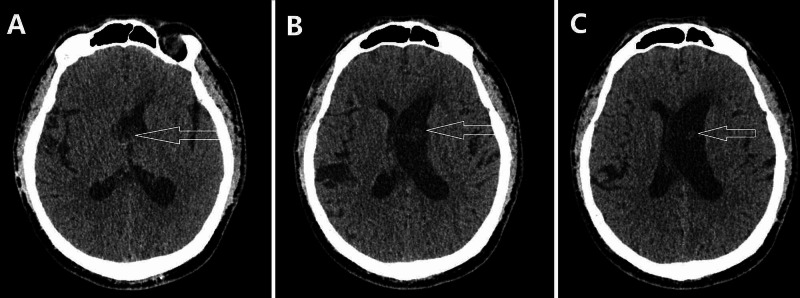
Axial computed tomography. Axial computed tomography scan revealing an intraventricular low-density lesion without a calcification mass attached to the anterior third of the septum pellucidum and Monro's foramen and enlargement of the left lateral ventricles.

**Figure 2:**
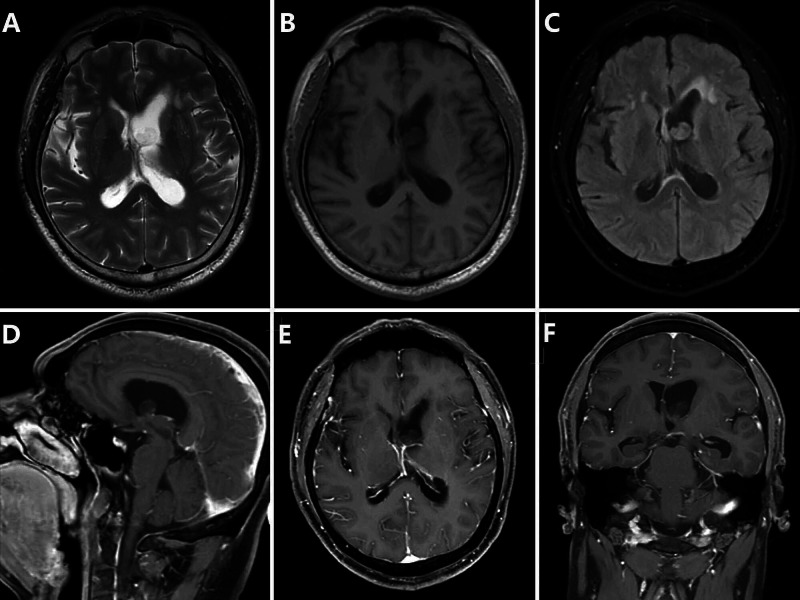
MRI of the patient. T2 and T2-FLAIR axial images demonstrating a clearly hyperintense lesion (A, C). T1-weighted axial image demonstrating isointensity relative to gray matter (B). T1-weighted axial image with gadolinium administration that did not reveal any enhancement (D, E, F). FLAIR: fluid-attenuated inversion recovery.

The preoperative laboratory results were normal, and the chest computed tomography (CT) scan showed no evidence of a pathology. Upon examination, the patient was found to be a little drowsy but was able to obey commands. His vital signs at the time of admission were as follows: blood pressure of 104/66 mmHg, pulse rate of 97 beats per minute, respiratory rate of 24 breaths per minute, and temperature of 36.7°C. His Glasgow coma scale score was 15/15. His vital signs were all within normal ranges. The rest of the neurological and respiratory examination findings were found to be unremarkable. A CT scan of the head showed an intraventricular mass attached to the anterior third of the septum pellucidum and Monro's foramen and enlargement of the left lateral ventricles (Figure 1). A magnetic resonance image confirmed an intraventricular cystic tumor 1.9 cm in diameter without enhancement with gadolinium (Figure 2). Furthermore, active left lateral ventricle hydrocephalus was present. A colloid cyst was considered the primary diagnosis.

With the patient in the supine position and the head turned 10 to 15 degrees to the opposite side, a curvilinear skin incision that was 12 cm long was made around Kocher's point, which was located 1 cm in front of the palpated coronal suture and 3 cm from the midline (along the midpupillary line). A craniotomy that was 5 cm long and 4 cm wide was performed to expose the main part of the middle frontal gyrus. After the cortical surface was coagulated, a soft catheter (Medtronic, Minneapolis, Minnesota, USA, #27703 or 26026) was used for the initial accurate ventricular entry. The catheter was aimed at the ipsilateral external auditory meatus in the coronal plane and the contralateral medial canthus in the sagittal plane. The cerebrospinal fluid (CSF) flow was visualized after the catheter was advanced 4 cm. After the operative corridor was created along this catheter, the left lateral ventricle was opened, and the tumor was revealed. The color of the tumor was milky white, and it appeared to be similar to a peeled lychee. Only a tiny vessel of the tumor adhered to the roof of the third ventricle. The tumor did not adhere to the fornix. The vessel was coagulated while care was taken to prevent heat damage or direct trauma to the fornix. The entire tumor wall was removed in a gross fashion. The same catheter was then left in the left ventricle. The surgery lasted two hours, and anesthesia was induced for three hours. The patient exhibited stable vital signs during the surgery.

After the operation, the patient was conscious and was admitted to the intensive care unit (ICU) at 1:00 p.m., where the patient did not suffer convulsions. Starting at 2 pm, the urine volume of the patient exceeded 250 ml per hour for 10 hours. At the 5th hour after the operation, the urine volume was as high as 700 ml. During the 10 hours, after the administration of 12 units of posterior pituitary injection (Anhui Hongye Pharmaceutical Co., Ltd), the patient's urine volume returned to normal. Since then, the patient's urine volume did not exceed 200 ml per hour.

At 8:00 p.m., a high fever occurred, and the patient’s body temperature reached 38.8°C. At 10:00 p.m., his body temperature reached 39.6°C. Physical cooling and antipyretic drugs were administered, showing favorable results. Six hours later, his temperature was normal, 37°C.

**Figure 3:**
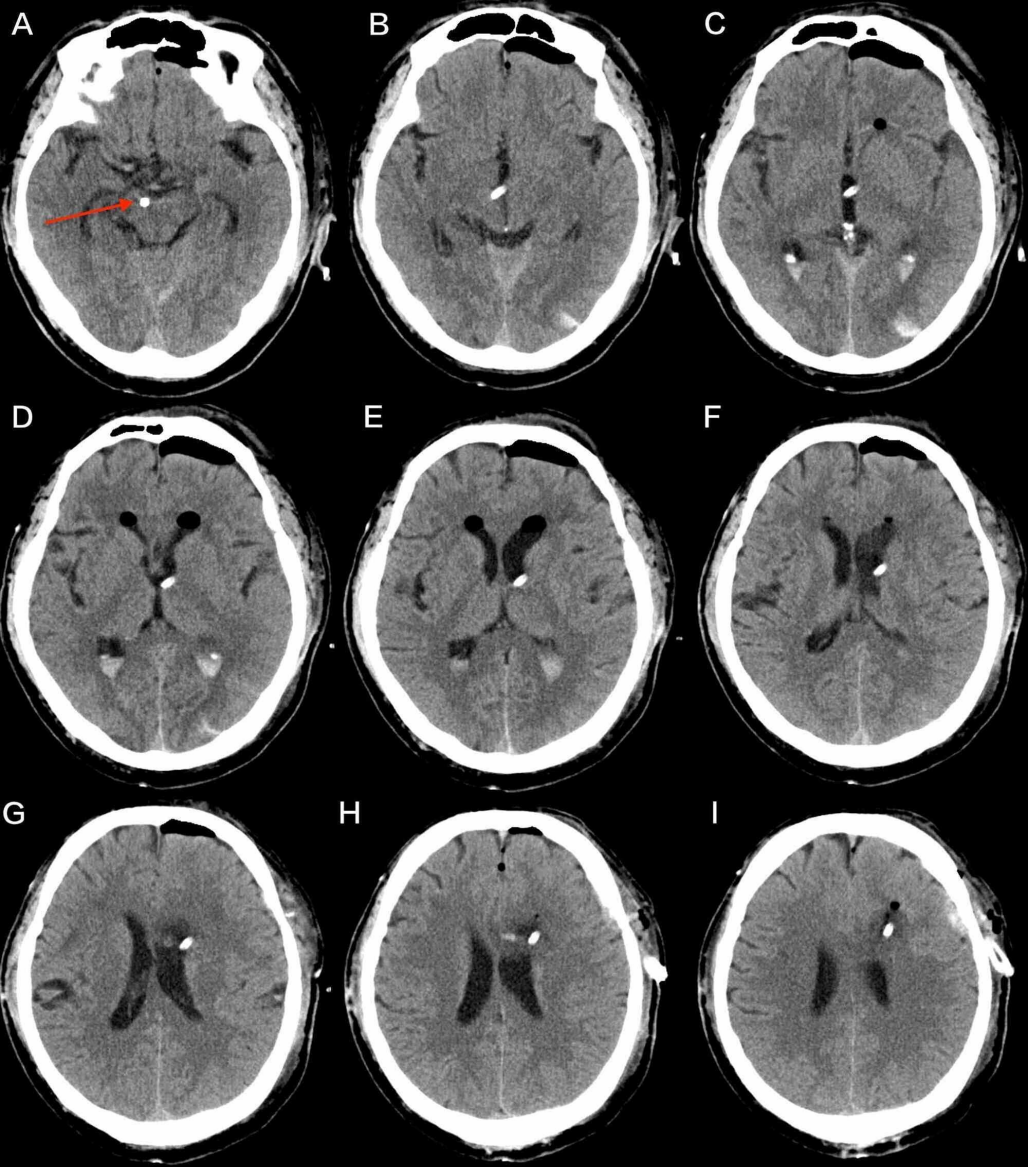
Axial CT scan (day 1 after the operation). Axial CT scan (day 1) revealing that the catheter traveled downward through the inferior parts of the third ventricle and reached the right cerebral peduncle (red arrowhead).

At the 19th hour after the operation, the head CT scan showed no intracranial hemorrhage, but the length of the catheter in the brain was close to 7 cm, and the tip of the catheter was located at the right cerebral peduncle (Figure 3).

At the 20th hour after the operation, the catheter was removed. On day 1 after surgery, the morning (8:00) ACTH level was 1.2 pg/ml (normal, 7.2-63.4 pg/ml), and the cortisol concentration was 0.09 µg/dL (normal, 4.26-24.85 µg/dL). Following stabilization, the patient was transferred to the general neurological ward. He was discharged home with no neurological deficits on day 11. The final histologic diagnosis was subependymoma of WHO grade I.

**Figure 4:**
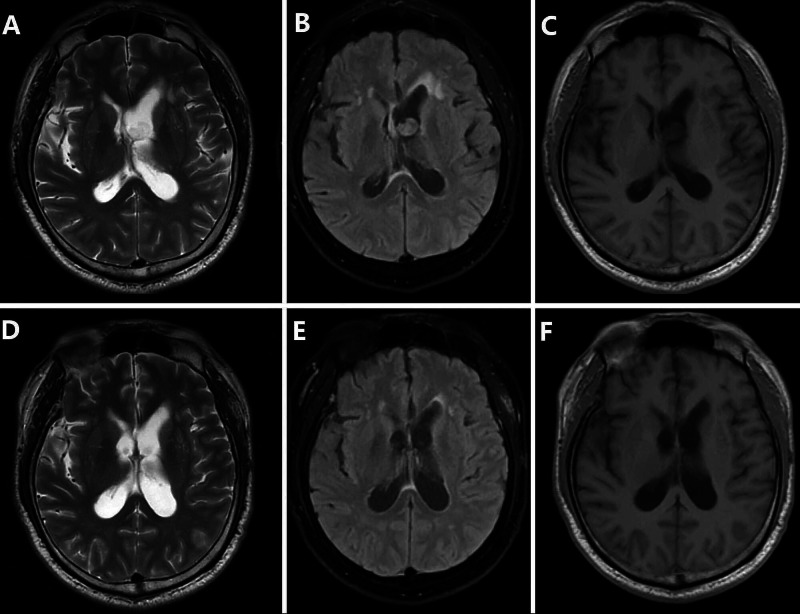
Pre- and post-operation MRI. Head magnetic resonance imaging scan before the operation showing the tumor (A, B, C); brain MRI scan at three months after the operation demonstrating a nearly normal lateral ventricle (D, E, F).

Two months later, his ACTH level was normal, and he never needed to take prednisone again (Figure 4). He has been working as a taxi driver again for three months and has not experienced headache, fatigue or dizziness.

## Discussion

The patient in our study suffered from central diabetes insipidus within just two hours after the operation and presented with hyperthermia within six hours after the operation. ACTH deficiency was evident on day 1 after surgery. All these clinical manifestations remind us that hypothalamic injury can occur. However, the tumor was located at a distance from his hypothalamus.

The hypothalamus forms the floor and inferior-lateral walls of the third ventricle and is located between the rostral midbrain and the lamina terminalis [3].

The underlying cause of central diabetes insipidus is the insufficient synthesis or inadequate secretion of arginine vasopressin (AVP) upon osmotic stimulation.

Central diabetes insipidus (CDI) is characterized by the insufficient synthesis or inadequate secretion of arginine vasopressin (AVP; also called antidiuretic hormones or ADHs), resulting in varying degrees of polyuria [4]. AVP is a neuropeptide produced by magnocellular neurons of the paraventricular and supraoptic nuclei [5]. AVP deficiency can be caused by any changes that take place at one or more of the sites involved in AVP excretion: the paraventricular or supraoptic nuclei; the hypothalamic osmoreceptors; or the superior portion of the supraopticohypophyseal tract [6]. Parvocellular neurons of the paraventricular nuclei containing corticotrophin-releasing hormones project to the hypophyseal portal system and induce the release of ACTHs [1].

Axial brain CT scan images taken 19 hours after the operation show the complete passage of the catheter. We can see that the tip of the catheter was located very close to the right cerebral peduncle. The catheter is usually inserted into the left lateral ventricle and usually remains there, allowing CSF to flow through it and into a drainage bag. However, our catheter traveled downward through the inferior parts of the third ventricle and reached the right cerebral peduncle. According to Figure 2, this catheter must have caused direct damage to the paraventricular nuclei of the hypothalamus. Clinical manifestations of hypothalamic damage include diabetes insipidus, disturbances in temperature regulation, death, coma, etc. [2]. On the other hand, hypothalamic injury is the most common causes of death in patients undergoing transsphenoidal operations [7]. Regarding the patient in our study, iatrogenic damage led to CDI, a lower ACTH level and hyperthermia. After we removed the catheter at 19 hours after the operation, the patient never had polyuria or high fever again.

In fact, diabetes insipidus and hyperthermia were transient in the patient in our study partly because the damage caused by the catheter was minor. The tips of catheters are soft and blunt, so they usually cannot cause sharp and severe damage to the brain. After we sutured the dura mater, we checked the length of the catheter left beneath the dura mater. The operation was performed without complications and with less bleeding than expected, even though we did not have a navigation system. Unfortunately, the patient in our study suffered from an iatrogenic hypothalamus injury. Every neurosurgical operation needs to be performed carefully and cautiously.

## Conclusions

We report a case of iatrogenic hypothalamic damage caused by a drainage catheter, which was associated with transient central insipidus diabetes, hyperthermia and ACTH deficiency. This conclusion of iatrogenic hypothalamic damage must be inferential, because in fact, we have no histopathological diagnosis of hypothalamic injury. This scenario has not been previously described in the literature. This unusual complication should be taken into account in patients who need external ventricular drains. Much attention should be paid to ensure that the length of the drainage catheter beneath the brain surface does not exceed 5 cm.
